# A Study on the Characterization of Asphalt Plant Reclaimed Powder Using Fourier Transform Infrared Spectroscopy

**DOI:** 10.3390/ma18153660

**Published:** 2025-08-04

**Authors:** Hao Wu, Daoan Yu, Wentao Wang, Chuanqi Yan, Rui Xiao, Rong Chen, Peng Zhang, Hengji Zhang

**Affiliations:** 1 Shandong Provincial Road and Bridge Group Co., Ltd., Jinan 250014, China; 2 School of Civil Engineering, Southwest Jiaotong University, Chengdu 610031, Chinaycq@swjtu.edu.cn (C.Y.); 3 Sichuan Transportation Construction Group Co., Ltd., Chengdu 610041, China; 4 Shanxi Transportation Holdings Science & Technology Transformation Co., Ltd., Taiyuan 030012, China

**Keywords:** reclaimed powder, infrared spectroscopy (FTIR), acidity/alkalinity, cleanliness

## Abstract

Asphalt plant reclaimed powder is a common solid waste in road engineering. Reusing reclaimed powder as filler holds significant importance for environmental protection and resource conservation. The key factors affecting the feasibility of reclaimed powder reuse are its acidity/alkalinity and cleanliness. Traditional evaluation methods, such as the methylene blue test and plasticity index, can assess reclaimed powder properties to guide its recycling. However, these methods suffer from inefficiency, strong empirical dependence, and high variability. To address these limitations, this study proposes a rapid and precise evaluation method for reclaimed powder properties based on Fourier transform infrared spectroscopy (FTIR). To do so, five field-collected reclaimed powder samples and four artificial samples were evaluated. Scanning electron microscopy (SEM), X-ray fluorescence spectroscopy (XRF), and X-ray diffraction (XRD) were employed to characterize their microphase morphology, chemical composition, and crystal structure, respectively. Subsequently, FTIR was used to establish correlations between key acidity/alkalinity, cleanliness, and multiple characteristic peak intensities. Representative infrared characteristic peaks were selected, and a quantitative functional group index (Is) was proposed to simultaneously evaluate acidity/alkalinity and cleanliness. The results indicate that reclaimed powder primarily consists of tiny, crushed stone particles and dust, with significant variations in crystal structure and chemical composition, including calcium carbonate, silicon oxide, iron oxide, and aluminum oxide. Some samples also contained clay, which critically influenced the reclaimed powder properties. Since both filler acidity/alkalinity and cleanliness are affected by clay (silicon/carbon ratio determining acidity/alkalinity and aluminosilicate content affecting cleanliness), this study calculated four functional group indices based on FTIR absorption peaks, namely the Si-O-Si stretching vibration (1000 cm^−1^) and the CO_3_^2−^ asymmetric stretching vibration (1400 cm^−1^). These indices were correlated with conventional testing results (XRF for acidity/alkalinity, methylene blue value, and pull-off strength for cleanliness). The results show that the Is index exhibited strong correlations (R^2^ = 0.89 with XRF, R^2^ = 0.80 with methylene blue value, and R^2^ = 0.96 with pull-off strength), demonstrating its effectiveness in predicting both acidity/alkalinity and cleanliness. The developed method enhances reclaimed powder detection efficiency and facilitates high-value recycling in road engineering applications.

## 1. Introduction

During asphalt mixture production, raw materials in asphalt plants undergo transportation, production, heating, and mixing processes. Due to the hot-mix nature of asphalt mixtures, a significant amount of heavily polluted dust particles is generated [1]. These particles are collected by the plant’s dust removal system and stored in designated containers. Typically, these particles have a size smaller than 0.075 mm and are referred to as reclaimed powder (or reclaimed dust) [2].

The current practice of stockpiling or landfilling reclaimed powder not only occupies valuable land resources but also reduces land-use efficiency while creating negative impacts on energy production, resource utilization, and environmental protection [3]. With the rapid expansion of highway infrastructure [4], failure to properly recycle this material poses significant challenges to sustainable development [5].

Ratnasamy Muniandy [6] investigated the influence of mineral powder on the performance of stone mastic asphalt (SMA). The study revealed that excessive use of powder significantly increases the stiffness of the mastic portion. However, when key parameters of powder—including density, gradation distribution, appearance, the hydrophilicity coefficient, and plasticity index—meet specified requirements, it can be effectively utilized in SMA applications.

Chi-Wei Chen’s research [7] demonstrated that reclaimed powder not only satisfies the quality standards for mineral powder but also exhibits stronger alkalinity, which enhances the bonding between asphalt and aggregates. The Pennsylvania Department of Transportation [8] conducted a study on six types of reclaimed powder, concluding that when the dust content is maintained below 2%, the impact on mixture performance remains negligible.

When utilized as a filler replacement, reclaimed powder must meet stringent requirements regarding its acidity/alkalinity and cleanliness. Research by Najib Mukhtar et al. [9] demonstrated that the pH characteristics of fillers significantly influence the adhesion between filler particles and asphalt binder in asphalt mixtures. Frederick M. Fowkes [10] further established that this interfacial bonding strength shows strong correlation with the material’s silica content, which serves as a key indicator of its acid–base properties.

The cleanliness of reclaimed powder, particularly its clay content, represents another crucial quality parameter. Wu Xuan et al. [11] found that impurities in filler materials can adversely affect the high-temperature performance of asphalt mixtures. Complementary research by Gajewski [12] revealed that excessive impurities may increase the optimum water content, leading to reduced binder viscosity [13], consequently impairing both the mechanical properties and long-term durability of the mixture.

Current evaluation methods for these parameters are specified in the National Standard “Rock Classification and Nomenclature Scheme”. The standard recommends X-ray fluorescence (XRF) spectroscopy for determining filler acidity/alkalinity, while cleanliness assessment primarily focuses on clay content through either methylene blue value (MBV) testing or plasticity index (PI) measurement [14].

Although these methods provide feasibility for testing reclaimed powder properties, they still present several problems. Microanalysis equipment like XRF is difficult to widely implement during construction processes; while methylene blue and plasticity index tests are relatively common, their testing procedures are overly complex, heavily empirical, require highly skilled operators, and show significant variability. These issues limit the large-scale, high-efficiency recycling applications of reclaimed powder. Hou [15] used XRF, noting that while XRF instruments have high sensitivity, their high cost and bulk size make them unsuitable for portable use. JM Moreno-Maroto [16] conducted an examination of fine-grained soil classification systems based on plasticity, with test results showing that plasticity index tests have clear deficiencies when testing particles of different minerals, requiring comprehensive judgment through other mineral composition analysis tests. SK Singh [17] and others researched detection methods for material clay content, with studies showing that the current standard methylene blue test method relies on assumed preconditions, making it quite empirical.

Currently, many field laboratories of China are equipped with portable infrared spectrometers for measuring SBS content [18]. These infrared spectrometers can rapidly detect the chemical composition of various materials, including fillers and reclaimed powder [19]. At the same time, the operation is simple and easy to carry out, which provides a hardware foundation for the rapid and accurate testing of the acidity/alkalinity and cleanliness of the reclaimed powder [20]. Given these circumstances, this paper proposes a new method for evaluating and classifying reclaimed powder from asphalt mixing plants based on Fourier transform infrared spectroscopy (FTIR) tests. This study uses infrared spectrometers to scan reclaimed powder, extract relevant functional group information, establishes correlations between this information and the acidity/alkalinity and cleanliness indicators of reclaimed powder, and ultimately guides the classification and recycling of reclaimed powder.

## 2. Materials and Methods

### 2.1. Materials

**Figure 1:**
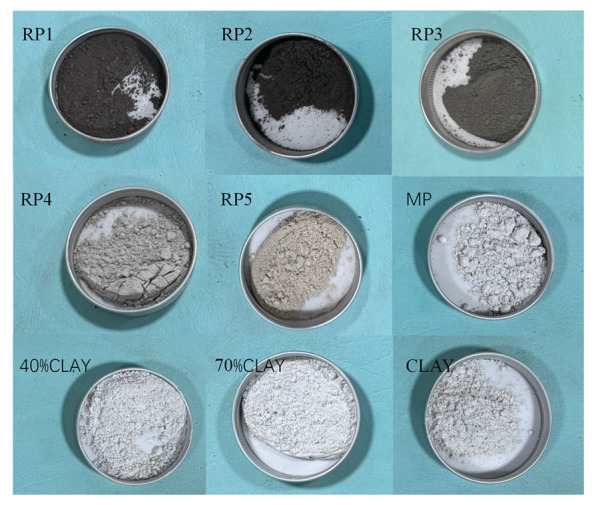
The powders used in this study.

To develop a Fourier transform infrared spectroscopy (FTIR)-based detection method for reclaimed powder, five types of reclaimed powder were collected from different fields, as shown in Figure 1. During collection, the damper opening of the mixing plant’s dust removal system was set to 32%, with a flue gas flow velocity of 13.8 m/s. For convenience in subsequent descriptions, the reclaimed powder samples are abbreviated as RP1 to RP5.

Given the significant variability in field-collected reclaimed powder, artificial reclaimed powder was prepared to obtain more trend-conclusive results. The artificial samples were created by artificially adding 40% and 70% clay to limestone mineral powder (MP), designated as 40% clay and 70% clay, respectively, to simulate different cleanliness levels. Additionally, pure clay was also tested for reference.

The basic properties of the nine powder materials were tested, and the results are shown in Table 1. The density and particle size distribution of the reclaimed powder were similar to those of mineral powder, but significant color variations were observed among the reclaimed powder samples. These color differences may be attributed to substantial variations in the mineral composition of aggregates used in different regions.

### 2.2. Methods

#### 2.2.1. Scanning Electron Microscopy (SEM)

To observe the micromorphology of the powder materials, scanning electron microscopy (SEM) analysis was conducted. SEM testing was performed using a JSM-IT500 instrument (JEOL, Kyoto, Japan) with an acceleration voltage of 20 kV. Prior to testing, the samples underwent a 5 min gold sputtering process under vacuum to enhance surface conductivity. This pretreatment prevents charge accumulation effects that could otherwise interfere with electron signal transmission and lead to image distortion, deformation, or instability.

#### 2.2.2. X-Ray Fluorescence Spectroscopy (XRF) Test

To determine the elemental composition of the reclaimed powder and subsequently evaluate its acidity/alkalinity, X-ray fluorescence (XRF) spectroscopy was performed on all nine powder samples. The XRF testing was conducted using a Shimadzu XRF-1800 spectrometer (Shimadzu, Kyoto, Japan) with the following specifications: rhodium target X-ray tube; maximum power output: 4 kW; window thickness: 75 μm; elemental detection range: oxygen (O) to uranium (U); and analysis mode: top irradiation geometry.

#### 2.2.3. X-Ray Diffraction (XRD) Test

X-ray diffraction (XRD) analysis was conducted to characterize the phase composition of the reclaimed powder and identify its crystalline species and structure. The XRD testing was performed using a Bruker D8 Advance diffractometer (Rigaku Corporation, Kyoto, Japan) with the following experimental parameters: scanning range: 10° to 90° (2θ); scanning speed: 2°/min; and step size: 0.02°.

#### 2.2.4. Fourier Transform Infrared Spectroscopy (FTIR) Test

Fourier transform infrared spectroscopy (FTIR) was employed to analyze the powder samples. The infrared spectrometer model was Nicolet iS20 FTIR (Thermo Fisher Scientific, Waltham, MA, USA). Using attenuated total reflection (ATR) accessories, the apodization function was Happ-Genzel, the number of scans was 64, and FTIR scanning was performed in the wavenumber range of 400–2000 cm^−1^.

**Figure 2:**
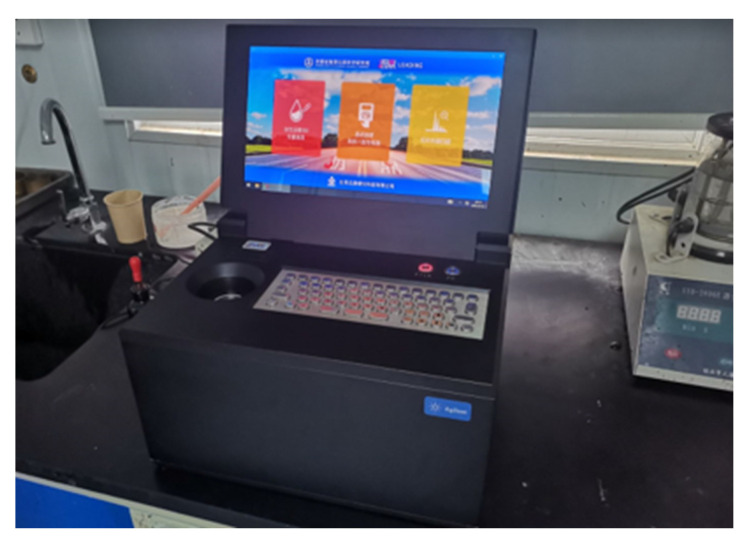
Portable infrared spectrometers.

Approximately 1 g of the sample was placed on the ATR crystal surface for spectral acquisition. The obtained spectra were subsequently processed using MATLAB software (R2024b) for quantitative analysis, from which evaluation indices for both acidity/alkalinity and cleanliness were extracted using portable infrared spectrometers, as shown in Figure 2.

#### 2.2.5. Methylene Blue (MB) Test

To evaluate the cleanliness of the reclaimed powder, methylene blue tests were conducted on the powder materials in accordance with the JTG 3432-2024 (Test Methods of Aggregates for Highway Engineering, Ministry of Transport of the People’s Republic of China, 2024) [21]. The standard methylene blue solution was prepared using analytically pure methylene blue crystals and distilled water, with titration performed on the suspension using quantitative filter paper. The test results were expressed as the methylene blue value (MBV) of the materials. The MBV primarily correlates with the content of fine particles having large specific surface areas (such as clay) in the filler.

## 3. Results and Discussion

### 3.1. SEM Observation Results (Filler Micromorphology)

**Figure 3:**
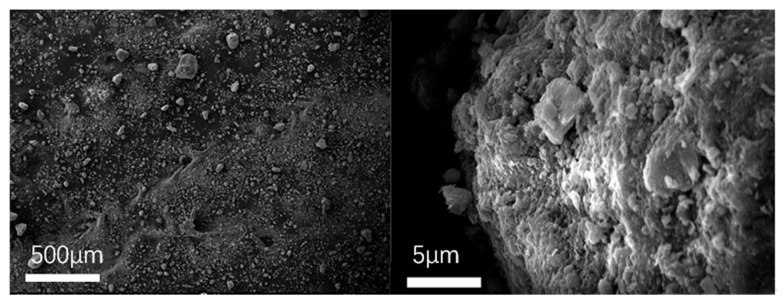
SEM micrograph of RP1.

**Figure 4:**
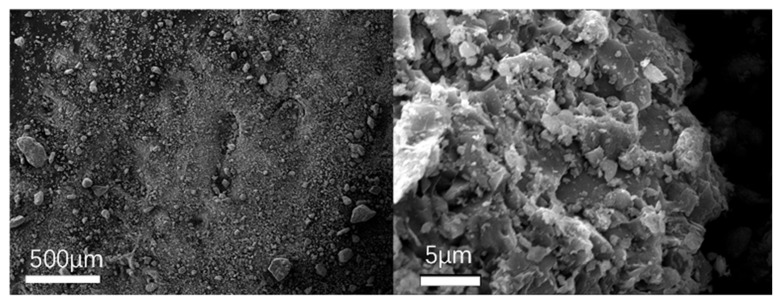
SEM micrograph of RP2.

**Figure 5:**
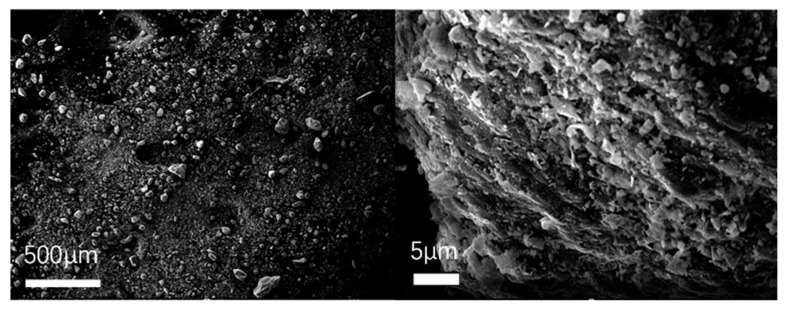
SEM micrograph of RP3.

**Figure 6:**
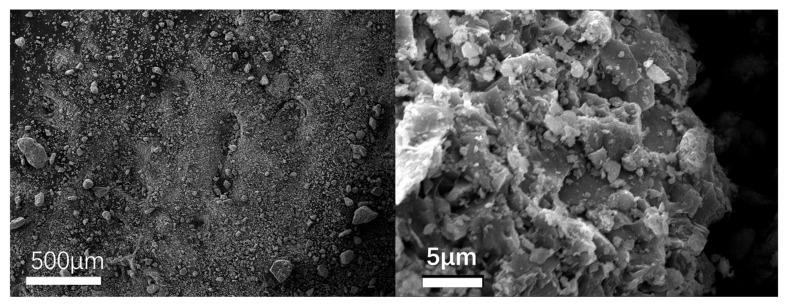
SEM micrograph of RP4.

**Figure 7:**
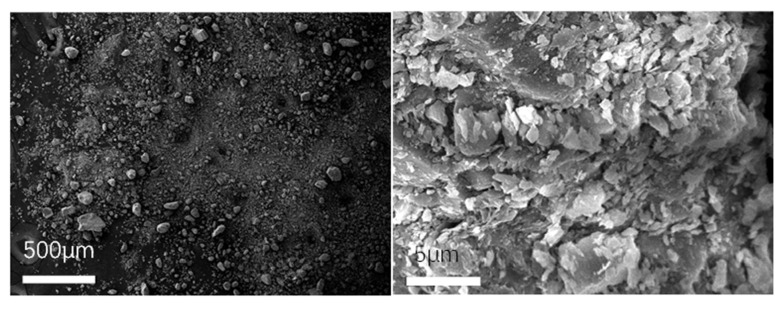
SEM micrograph of RP5.

To observe the micromorphology of various reclaimed powder samples, SEM scanning was performed to capture microscopic images under different magnification conditions. The SEM results for different reclaimed powders are presented in Figure 3, Figure 4, Figure 5, Figure 6 and Figure 7. The left panel of each figure shows the SEM image at 50×, while the right panel displays a locally magnified view at 2000×, with the scale bar positioned at the bottom left corner.

The images reveal that the reclaimed powder consists of accumulated fine particles approximately 1 μm in size. Most particles exhibit irregular flaky and flocculent morphologies, with a minority showing porous structures and spherical shapes. According to research by HE Bergna [22], the spherical particles are primarily composed of silicon oxide. Overall, the reclaimed powder demonstrates small particle sizes, significant variability, and distinct micromorphological differences across samples from different regions.

**Figure 8:**
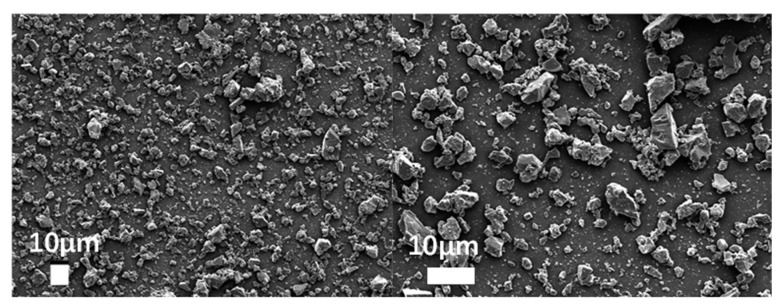
SEM micrograph of MP.

**Figure 9:**
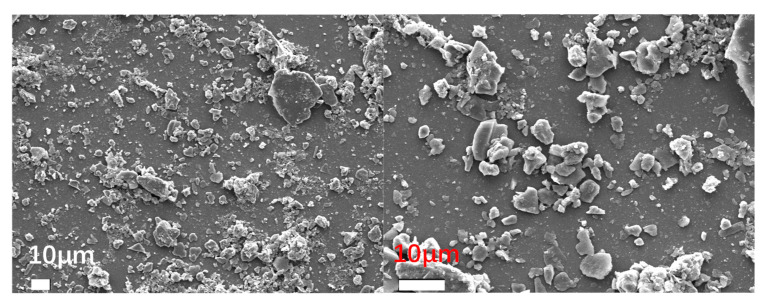
SEM micrograph of soil.

Based on the sources of the reclaimed powder, its primary components were determined to be mineral powder and dust particles. To better characterize the micromorphological features of the reclaimed powder, supplementary SEM images of mineral powder and soil were obtained for comparison (Figure 8 and Figure 9).

The mineral powder exhibited more uniform morphology with larger particles, while the soil particles appeared more irregular—a characteristic potentially attributable to the presence of organic matter in the soil. Comparative analysis revealed that the reclaimed powder shared morphological similarities with both reference materials, suggesting its composition likely consists of fragmented rock particles and dust.

### 3.2. XRF Test Results (Filler Acidity/Alkalinity)

XRF testing was conducted to determine the compound contents in the nine powder materials, with the measured compound contents shown in Table 2. The data for mineral powder, 40% clay, 70% clay, and pure clay showed linear correlations. RP1, RP2, and RP3 contained relatively higher contents of calcium carbonate (CaCO_3_), silicon oxide (SiO_2_), and iron oxide (Fe_2_O_3_), along with minor amounts of other compounds including aluminum oxide (Al_2_O_3_) and potassium oxide (K_2_O). In contrast, RP4 and RP5 were predominantly composed of calcium carbonate.

**Figure 10:**
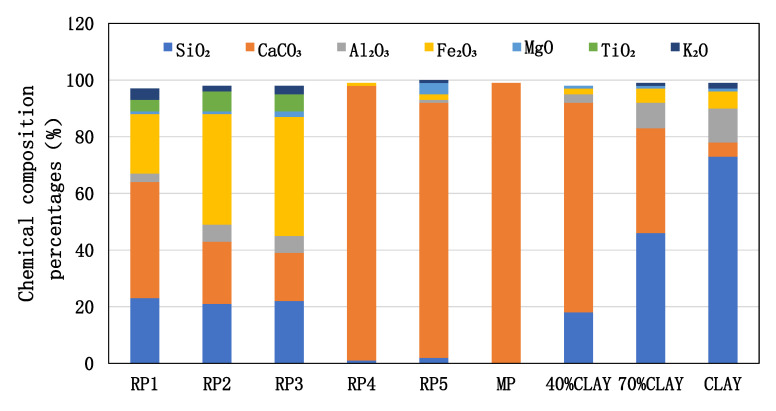
XRF results of chemical composition for nine powders.

The compound contents of the nine powder materials are shown in Figure 10. It can be observed that RP4, RP5, and mineral powder have relatively simple compositions, consisting mainly of calcium carbonate (CaCO_3_), while RP1, RP2, and RP3 contain higher amounts of impurities such as silicon oxide (SiO_2_) and iron oxide (Fe_2_O_3_). These results demonstrate significant variability in the properties of reclaimed powder, indicating the necessity of property testing prior to use. RP1, RP2, and RP3 were collected from municipal projects, whereas RP4 and RP5 came from highway projects. This indicates that construction quality control also plays an important role in ensuring the property consistency of reclaimed powder.

On the other hand, the silicon oxide (SiO_2_) content in mineral powder, 40% clay, 70% clay, and pure clay showed a linear increase, which was directly proportional to the clay content in these four materials. The SiO_2_ content exceeded 70% in pure clay. Higher SiO_2_ content in the powder materials resulted in lower alkalinity. It can be concluded that the addition of clay not only increases the acidity of the filler but also reduces its cleanliness. Therefore, for reclaimed powder, special attention must be paid to controlling the clay content. In general, when selecting aggregates, aggregates with high calcium carbonate content are called alkaline aggregates, and aggregates with high silica content are called acidic aggregates. The interaction between calcium carbonate and asphalt is stronger than that of silicon oxide, because calcium carbonate is a polar molecule, while silicon oxide crystal is a symmetrical atomic crystal composed of atoms. Therefore, when interacting with polar polymer asphalt, polar calcium carbonate exhibits stronger interaction [23,24]. This also explains the phenomenon that an alkaline aggregate has good adhesion to asphalt.

Since clay primarily consists of aluminosilicates, subsequent FTIR analysis may utilize silicon-related characteristic peaks (such as the Si-O-Si absorption peak at 1000 cm^−1^) to characterize clay presence and evaluate both the acidity/alkalinity and cleanliness of the filler materials.

### 3.3. XRD Test Results (Filler Crystal Structure)

**Figure 11:**
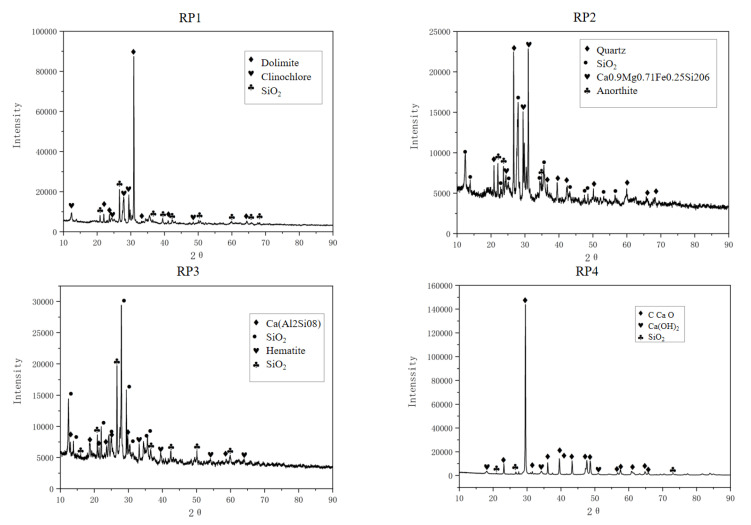

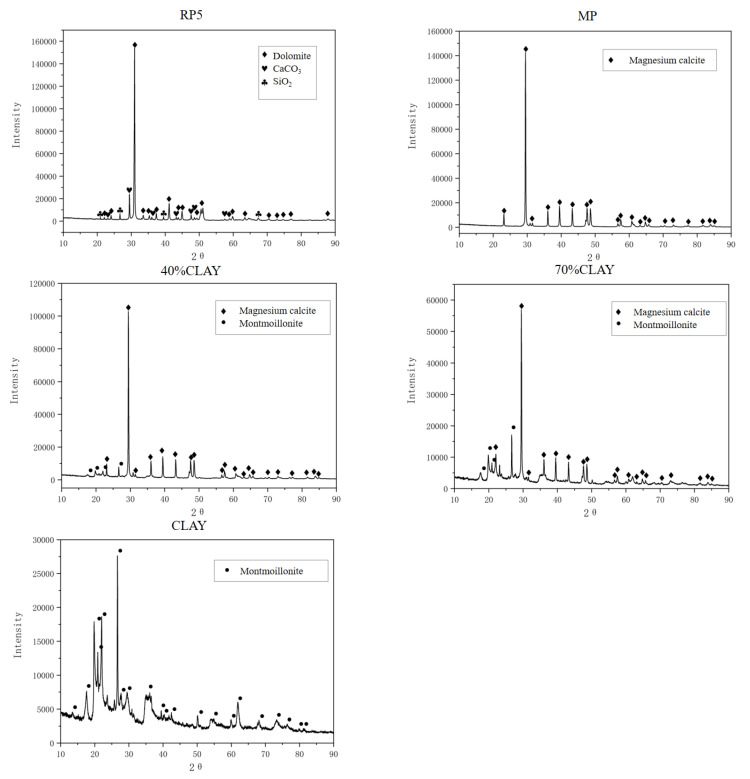
XRD spectra results of nine powders.

X-ray diffraction (XRD) analysis was conducted to examine the crystallization characteristics and crystal types of compounds in the nine powder materials, with the results shown in Figure 11. The diffraction intensities of various crystals exhibited a linear distribution that corresponded well to the formulation ratios of the four material types. The mineral powder displayed single absorption peaks, indicating its simple composition, while more complex waveforms suggested higher impurity content in other samples.

Peak fitting analysis revealed that the mineral powder had a relatively simple crystalline composition, predominantly calcite (primary component: calcium carbonate, CaCO_3_). As the clay content increased, the spectra became progressively more complex with additional impurity peaks, indicating higher clay impurity content primarily composed of aluminosilicates. All five reclaimed powder samples exhibited sharp characteristic peaks, demonstrating their highly crystalline nature. The main crystalline phases in the reclaimed powder were calcium carbonate (CaCO_3_) and silicon oxide (SiO_2_) crystals, including accessory minerals such as anorthite, quartz, diopside, and chlorite, along with various metal oxides. Compared to RP4 and RP5, samples RP1, RP2, and RP3 showed more impurity peaks, reflecting their more complex composition and higher impurity content, which is consistent with the XRF experimental results.

### 3.4. Methylene Blue Test Results (Filler Cleanliness)

The methylene blue (MB) test was employed to evaluate the cleanliness of fillers, primarily reflecting the content of fine particles with large specific surface areas (e.g., clay) in the filler materials. A higher MB value (MBV) indicates poorer cleanliness, while a lower MBV suggests cleaner material. To investigate the cleanliness of the reclaimed powder, MB tests were conducted on all nine powder samples, with the results presented in Table 3.

The MBVs of mineral powder, 40% clay, 70% clay, and pure clay exhibited a linear relationship, confirming the significant influence of clay content on material cleanliness. Due to substantial variations in particle composition and impurity levels among reclaimed powders from different sources, their MBVs showed considerable differences. RP4 and RP5 demonstrated better cleanliness, whereas RP1, RP2, and RP3 had higher MBVs, highlighting the importance of construction quality control.

Notably, RP4 showed a similar MBV to mineral powder, which can be combined with previous test results indicating their analogous calcium carbonate (CaCO_3_) composition. This suggests that RP4 could potentially be a substitute for mineral powder in recycling applications, despite minor micromorphological differences.

### 3.5. FTIR Spectroscopy Analysis

#### 3.5.1. Functional Group Analysis and Characteristic Peak Index Calculation of Fillers

**Figure 12:**
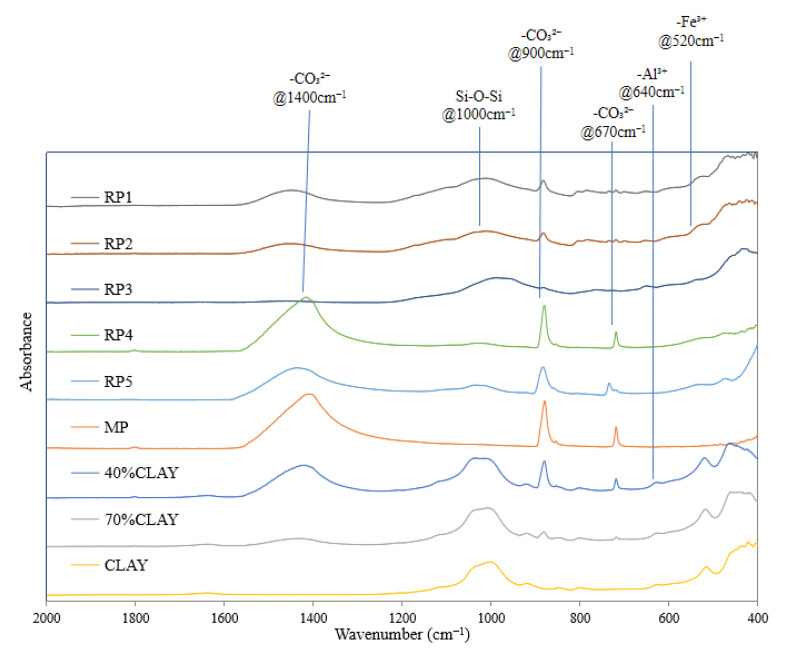
FTIR spectra of nine powders.

The XRD and XRF instruments mentioned above are primarily research equipment and are hard to access in a field lab, whereas infrared spectroscopy, as a more common testing instrument, can be equipped in field laboratories. FTIR spectroscopy offers multiple advantages for powder analysis including a fast scanning speed, simple procedures, and accurate results. Therefore, this study proposes using functional groups in infrared spectra as indicators to evaluate both the cleanliness and acidity/alkalinity of powder materials. The preliminary SEM and XRD characterizations provide a morphological reference for the recycled powder, which is beneficial to determine the appropriate FTIR scanning range and characteristic peak selection. To analyze the functional groups related to these properties in the reclaimed powder, FTIR scans were performed on all nine samples. Each homogenized sample underwent triplicate tests under identical conditions to verify spectral reproducibility, with results showing high consistency across replicates. Since no absorption peaks were observed at wavenumbers above 2000 cm^−1^, the spectral range of 400–2000 cm^−1^ was selected for data presentation, with the results shown in Figure 12.

An analysis of Figure 12 with reference to related studies [25] identifies the characteristic functional group peaks as follows: the broad peak at 1000 cm^−1^ corresponds to the silicon–oxygen–silicon (Si-O-Si) stretching vibration, while Buzgar Nicolae et al. [26] confirmed that the peak at 1400 cm^−1^ represents the antisymmetric stretching vibration of carbonate groups (-CO_3_^2−^). Additional carbonate-related peaks include the out-of-plane bending vibration at 900 cm^−1^ and the in-plane bending vibration at 670 cm^−1^. Furthermore, another study [27] assigned the peaks at 520 cm^−1^ and 640 cm^−1^ to trivalent iron (-Fe^3+^) and aluminum ions (-Al^3+^), respectively.

The analysis reveals a clear linear relationship between clay content and FTIR absorbance intensities for both carbonate (CO_3_^2−^) and siloxane (Si-O-Si) functional groups across the four reference samples (mineral powder, 40% clay, 70% clay, and pure clay). Specifically, RP4 and RP5 exhibit strong carbonate peaks (1400, 900, 670 cm^−1^) with minimal Si-O-Si absorption (1000 cm^−1^); RP3 shows pronounced peaks for both functional groups; and RP1 and RP2 demonstrate moderate absorbance for both peaks. These spectral patterns align with previous XRF, XRD, and MBV test results, confirming the consistency of our multi-technique characterization approach for evaluating reclaimed powder properties.

The experimental results demonstrate that the addition of clay simultaneously increases filler acidity and reduces cleanliness, necessitating strict control of clay content in reclaimed powder applications. Since aluminosilicates constitute the primary components of clay, a quantitative assessment of clay content can be achieved through characterizing the Si-O-Si absorption peak at 1000 cm^−1^ in the FTIR spectra.

The filler composition, predominantly comprising silicon and calcium components, allows for a quantitative assessment using characteristic carbonate absorption peaks observed at 1400 cm^−1^ (strong and broad antisymmetric stretching vibration), 900 cm^−1^ (weak out-of-plane bending vibration), and 670 cm^−1^ (weak in-plane bending vibration). The 1400 cm^−1^ peak proves particularly advantageous for quantitative analysis due to its high intensity, broad spectral window, and absence of overlapping interference from other functional groups. While the smaller 900 cm^−1^ and 670 cm^−1^ peaks are also indicative of carbonate content, they present challenges in differentiation and quantification due to their lower intensities and potential spectral overlaps. This spectral characterization approach enables precise carbonate content determination through 1400 cm^−1^ peak area integration.

**Figure 13:**
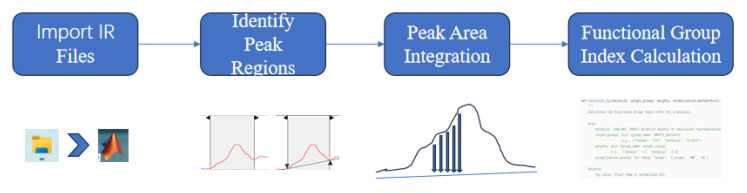
Schematic of spectral data processing steps.

Based on the aforementioned analysis, the antisymmetric stretching vibration peak of carbonate at 1400 cm^−1^ (hereinafter referred to as anti-carbonate) and the silicon–oxygen–silicon (Si-O-Si) absorption peak at 1000 cm^−1^ were selected from multiple functional group absorption peaks as the characteristic peaks in the infrared spectrum of reclaimed powder, serving as indicators for material acidity/alkalinity and cleanliness. Furthermore, in Fourier transform infrared spectroscopy, two common approaches are employed, namely a direct comparison of peak intensities and a semi-quantitative analysis using peak area ratios. Semi-quantitative analysis does not introduce new functional groups; it eliminates the influence of the sample amount on the spectral amplitude by the ratio [28]. The ratio of these two peak intensities (Si-O-Si to anti-carbonate and anti-carbonate to Si-O-Si) was adopted as an additional indicator for acidity/alkalinity. Since the area under the peak is a quantitative characterization of the functional group concentration standard, MATLAB programs were utilized to process the spectral data and automatically calculate functional group indices, with the processing workflow illustrated in Figure 13 and the calculation formulas provided in Equations (1)–(4). The results are presented in Table 4, where the zero value indicates concave spectral features in the corresponding regions, suggesting the absence of detectable absorption peaks. Subsequently, this study optimized the four quantitative indicators based on actual test results from XRF, pull-off tests, and methylene blue tests, ultimately proposing the evaluation indicator with the best predictive performance.

(1) $$I_{C}=A_{1400}$$

(2) $$I_{S}=A_{1000}$$

(3) $$I_{S/C}=\frac{A_{1000}}{A_{1400}}$$

(4) $$I_{C/S}=\frac{A_{1400}}{A_{1000}}$$

Nomenclature:

*I_C_*: Carbonate functional group index (1310 cm^−1^~1550 cm^−1^);
*I_S_*: Siloxane functional group index (930 cm^−1^~1070 cm^−1^);
*I*_*S*/*C*_: Siloxane-to-carbonate ratio index;
*I*_*C*/*S*_: Carbonate-to-siloxane ratio index;
*A*_XXX_: Integrated peak area at XXX cm^−1^ wavenumber.

#### 3.5.2. Optimization of Evaluation Indicators for Acidity/Alkalinity (Based on XRF and Pull-Off Tests)

**Figure 14:**
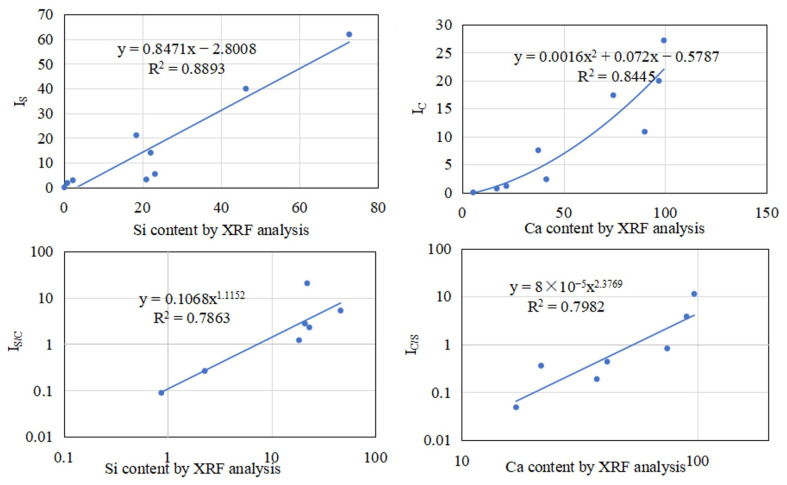
Correlation analysis between FTIR indices (I_S_, I_C_, I_S/C_, and I_C/S_) and corresponding XRF results.

To verify the correlation between the acidity/alkalinity indicators extracted from the FTIR tests and the actual material properties, a correlation analysis was conducted between the calculated functional group indices and XRF results, with I_C_ and I_S_ analyzed in standard Cartesian coordinates, while I_S/C_ and I_C/S_ were evaluated in logarithmic coordinates. The correlation analysis mainly focuses on empirical formula fitting to establish a predictive relationship for the performance of the recycled powder [29], as shown in Figure 14.

Except for I_S/C_, which showed a weaker correlation with XRF data, the other three indices (I_C_, I_S_, and I_C/S_) all exhibited quite good correlations with the filler’s actual acidity/alkalinity, particularly I_C_ and I_S_, which achieved superior correlation coefficients (R^2^ = 0.8893 and 0.8445, respectively). Therefore, they were selected for subsequent analysis.

The acidity/alkalinity of fillers significantly influences the adhesive properties of asphalt mastics. The reason for this phenomenon may be that the surface of clay minerals rich in hydroxyl (-OH) adsorbs water molecules and forms an interfacial water film with a thickness of 5–10 nm. This water film physically separates the asphalt from the aggregate, thereby reducing the adhesion of the asphalt [30].

**Figure 15:**
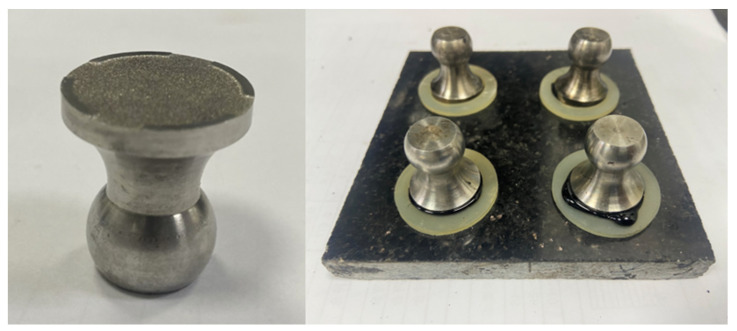
Pull-off test pull-head configuration and test specimen.

Therefore, in addition to XRF testing, pull-off tests were conducted on asphalt mastics incorporating different reclaimed powders. ESSO 70# asphalt (produced by ExxonMobil, Houston, TX, USA) was heated to 150 °C and mixed with nine types of powder materials at a 25% filler content. The fluid asphalt mastic was then dropped onto a basalt aggregate substrate, covered with a pull-head, and subjected to controlled pressure. The pull-head design featured a 0.2 m protruding edge at the bottom to serve as a support, allowing for excessive asphalt mastic to flow out through overflow channels. The pull-head configuration for the pull-off test is illustrated in Figure 15.

**Figure 16:**
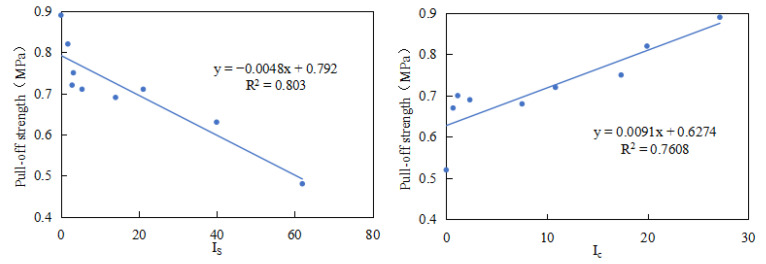
Correlation between FTIR index (I_S_, I_C_) and pull-off strength.

The specimens were then cured in a constant-temperature chamber at 25 °C for 2 h before being tested using a Positest AT-A pull-off adhesion tester, which applied vertical tension to the pull-heads bonded to the stone substrates. The pull-off strength, recorded after adhesive failure, served as an indicator of both the cohesive and adhesive properties of the asphalt, with each sample tested in triplicate and the results averaged. Subsequent correlation analysis between the FTIR-derived functional group indices (I_C_ and I_S_) and the pull-off strength (Figure 16) revealed strong relationships (R^2^ = 0.7608 for I_C_ and 0.803 for I_S_), demonstrating that the acidity/alkalinity indicators obtained through infrared spectroscopy effectively correlate with the bonding performance of asphalt mastics. These findings confirm that FTIR spectroscopy can reliably predict both the chemical properties (acidity/alkalinity) and macroscopic mechanical behavior (adhesion) of fillers, providing a rapid assessment method for quality control in practical applications.

#### 3.5.3. Optimization of Cleanliness Evaluation Indicators (Based on Methylene Blue Test)

**Figure 17:**
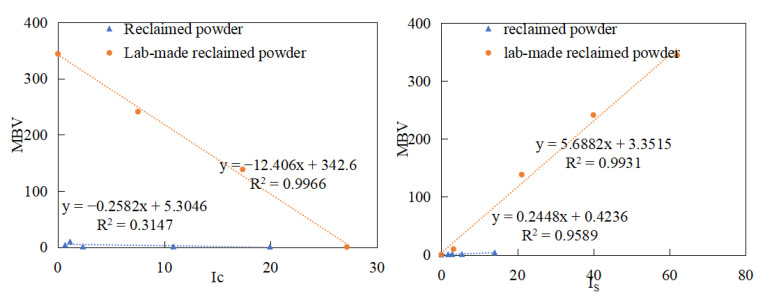
Correlation analysis of FTIR indices (I_S_, I_C_) and methylene blue values.

The evaluation indicators for acidity/alkalinity demonstrated that both I_C_ and I_S_ exhibited good correlation with pH properties (R^2^ > 0.84). This section further evaluates their effectiveness for cleanliness evaluation by conducting correlation analyses between I_C_ and I_S_ and MBV. The results are shown in Figure 17.

The analysis first reveals that the addition of clay significantly increases methylene blue values (MBVs) (orange data points in the figure), demonstrating its substantial impact on cleanliness. While the I_S_ index shows strong predictive capability for both clay-rich samples and conventional reclaimed powder samples, the I_C_ index performs well only for high-clay-content samples but performs poorly for standard reclaimed powders, indicating limited sensitivity. Given that field-collected reclaimed powders are more representative of real-world conditions, the I_S_ index is recommended as the preferred predictor for practical applications.

The results from the methylene blue tests, XRF analysis, and pull-off tests collectively demonstrate that the I_S_ index effectively predicts both the acidity/alkalinity and cleanliness of fillers, which can be attributed to the aluminosilicate composition of clay—while filler acidity/alkalinity is primarily influenced by silicon oxide (SiO_2_) content and cleanliness is governed by clay content, both parameters fundamentally correlate with the silicon content in fillers, thereby making the Is index (quantifying Si-O-Si functional groups at 1000 cm^−1^) a robust predictive indicator for these two critical properties, as evidenced by its strong correlations with SiO_2_ content (R^2^ = 0.87), MBV (R^2^ = 0.79) and pull-off strength (R^2^ = 0.81), confirming the dual diagnostic capability of this FTIR-derived spectral index for the simultaneous quality assessment of reclaimed powder materials.

## 4. Conclusions

To improve reclaimed powder utilization from asphalt plants, this study analyzed five field-collected and four artificial samples using SEM/XRF/XRD to characterize their properties. FTIR was then used to establish correlations between key parameters (acidity/alkalinity and cleanliness) and specific peak intensities, developing functional group indices validated by XRF, methylene blue, and pull-off tests. The main conclusions are as follows:

1. The reclaimed powder mainly consists of tiny, crushed stone particles and dust, with significant variations in crystal structure and chemical composition, including calcium carbonate, silicon oxide, iron oxide, aluminum oxide, etc. Some samples also contain clay, which is the primary factor affecting their properties. The presence of clay increases the acidity of the filler while reducing its cleanliness. Since the main component of clay is aluminosilicate, the silicon–oxygen–silicon (Si-O-Si) characteristic peak at 1000 cm^−1^ can be used to quantitatively evaluate clay content.
2. In the infrared spectrum, the Si-O-Si absorption peak at 1000 cm^−1^ and the antisymmetric stretching peak of carbonate at 1400 cm^−1^ show excellent correlation with the calcium carbonate and silicon oxide content in reclaimed powder. These peaks can be used to predict the filler’s calcium carbonate content, clay content, acidity/alkalinity, and cleanliness. Higher calcium carbonate and lower silicon oxide content indicate less clay, stronger alkalinity, and better cleanliness, making the material more suitable for reuse.
3. Based on the Si-O-Si peak at 1000 cm^−1^ and the carbonate antisymmetric stretching peak at 1400 cm^−1^, four functional group indices (I_C_, I_S_, I_S/C_, I_C/S_) were calculated. Their predictive performance for acidity/alkalinity and cleanliness was compared using XRF, methylene blue tests, and pull-off tests, ultimately selecting the I_S_ index due to its strong correlation. The I_S_ index exhibited correlations of R^2^ = 0.89 with XRF, R^2^ = 0.80 with methylene blue values, and R^2^ = 0.96 with pull-off strength, confirming its effectiveness in predicting both acidity/alkalinity and cleanliness.
4. Methylene blue tests also revealed that, in the absence of clay, dust and organic residues are the main factors influencing the MBV. However, even trace amounts of clay cause a sharp increase in the MBV, showing a completely different trend compared to clay-free samples. Therefore, during transportation and storage, reclaimed powder should be prevented from contact with clay.

## Figures and Tables

**Table 1:** Basic parameters of reclaimed powders.

Property	Test Method	RP1	RP2	RP3	RP4	RP5	MP	40% CLAY	70% CLAY	CLAY
Density (g/cm^3^)	JTG 3432-2024	2.42	2.51	2.13	2.39	2.33	2.41	2.43	2.44	2.47
Particle size (nm)	Laster particle test	2946	3237	2891	2973	2976	3019	2985	3022	2926
Color	\	black	black	black	white	white	brown	brown	brown	brown
Source		municipal	municipal	municipal	highway	highway	lab-made	lab-made	lab-made	lab-made

**Table 2:** Chemical composition of nine powder materials (%).

Sample	SiO_2_	CaCO_3_	Al_2_O_3_	Fe_2_O_3_	MgO	TiO_2_	K_2_O	Total
RP1	23	41	3	21	1	4	4	98
RP2	21	22	6	39	1	7	2	98
RP3	22	17	6	42	2	6	3	98
RP4	1	97	0	1	0	0	0	99
RP5	2	90	1	2	4	0	1	100
MP	0	99	0	0	0	0	0	100
40% CLAY	18	74	3	2	1	0	0	100
70% CLAY	46	37	9	5	1	0	1	99
CLAY	73	5	12	6	1	0	2	99

**Table 3:** Methylene blue test results of nine powders.

Test Material	MBV
RP1	1.25
RP2	10
RP3	4
RP4	1
RP5	1.25
MP	0.5
40% CLAY	138.5
70% CLAY	241.25
CLAY	344.25

**Table 4:** Functional group indices of powders extracted via MATLAB semi-quantitative analysis.

	I_C_	I_S_	I_S/C_	I_C/S_
RP1	2.35	5.40	2.30	0.44
RP2	1.14	3.18	2.79	0.36
RP3	0.68	14.01	20.60	0.05
RP4	19.93	1.77	0.09	11.26
RP5	10.85	2.85	0.26	3.81
MP	27.17	0	0	/
40% CLAY	17.4	21.1	1.21	0.82
70% CLAY	7.5	39.9	5.32	0.19
CLAY	0.0	61.9	/	0.0

## Data Availability

The original contributions presented in this study are included in the article. Further inquiries can be directed to the corresponding authors.
